# Efficacy, toxicity, and prognostic factors of rituximab plus cyclophosphamide, doxorubicin, vincristine, and prednisone in elderly patients with diffuse large B-cell lymphoma: a single-center retrospective cohort study

**DOI:** 10.3389/fonc.2026.1785720

**Published:** 2026-04-13

**Authors:** Jin Zhao, Li Ma, Meijing Zheng, Xiaojing Guo, Xiaolian Wen, Liping Su

**Affiliations:** 1 Department of Hematology, Shanxi Province Cancer Hospital/ Shanxi Hospital Affiliated to Cancer Hospital, Chinese Academy of Medical Sciences/Cancer Hospital Affiliated to Shanxi Medical University, Taiyuan, Shanxi, China; 2 Shanxi Provincial Key Laboratory of Lymphoma Precision Diagnosis and Treatment Research, Taiyuan, Shanxi, China

**Keywords:** prednisone, diffuse large B-cell lymphoma, doxorubicin, elderly patients, prognostic factors, relative dose intensity, rituximab plus cyclophosphamide, treatment tolerability

## Abstract

**Background:**

Optimizing treatment for elderly patients with diffuse large B-cell lymphoma (DLBCL) remains clinically challenging because curative intent must be balanced against treatment-related toxicity and reduced treatment tolerance. This study aimed to evaluate the efficacy and toxicity of first-line rituximab plus cyclophosphamide, doxorubicin, vincristine, and prednisone (R-CHOP)-based treatment in elderly patients with DLBCL and to identify prognostic factors associated with overall survival.

**Methods:**

We conducted a single-center retrospective cohort study of 162 patients aged 60 years or older with newly diagnosed DLBCL who received first-line R-CHOP-based treatment. The main outcomes were overall survival, treatment delivery, and treatment-related toxicity. Relative dose intensity (RDI) was used to assess treatment delivery, and survival was analyzed using Kaplan–Meier methods and Cox proportional hazards regression. A study-specific geriatric-treatment delivery risk model was further evaluated and compared with the International Prognostic Index (IPI) using discrimination analysis.

**Results:**

Patients older than 80 years had a significantly lower median RDI than those aged 60 to 80 years (78.2% vs. 93.6%, P = 0.009) and a markedly higher treatment-related mortality rate (33.3% vs. 1.9%, P = 0.015). In multivariable analysis, RDI below 85% (hazard ratio [HR], 2.45; P< 0.001) and albumin below 35 g/L (HR, 2.03; P< 0.001) were independently associated with worse overall survival. The geriatric-treatment delivery risk model showed better discriminative performance than the conventional IPI, with a concordance index of 0.748.

**Conclusions:**

In elderly patients with DLBCL, survival outcomes were closely associated with treatment tolerance and baseline nutritional status. These findings suggest that supportive strategies aimed at preserving treatment delivery, rather than empiric age-based dose reduction alone, may improve clinical outcomes in appropriately selected patients.

## 1 Introduction

Diffuse large B-cell lymphoma (DLBCL) is the most common aggressive lymphoma encountered in clinical practice (1, 2). Because the incidence of DLBCL rises with age, a substantial proportion of patients are diagnosed in later life, when treatment decisions are often complicated by comorbidity, physiologic vulnerability, and marked heterogeneity in functional reserve (3, 4). Despite these challenges, rituximab-based anthracycline-containing immunochemotherapy remains the standard first-line treatment with curative intent for most fit patients, according to both international guidelines and routine clinical practice (5, 6). Among available regimens, rituximab plus cyclophosphamide, doxorubicin, vincristine, and prednisone (R-CHOP) continues to serve as the principal reference regimen for frontline treatment. At the same time, the emergence of novel agents and alternative treatment strategies has made treatment selection in elderly patients increasingly dependent on a careful balance between antitumor efficacy and treatment tolerability (7–10).

In real-world elderly populations, however, the effectiveness of R-CHOP is frequently limited not by insufficient antitumor activity alone, but by the difficulty of delivering treatment safely and consistently. Toxicity-driven dose reduction, treatment delay, and premature discontinuation remain common and may compromise curative potential (11). Among treatment-related complications, febrile neutropenia, severe infection requiring hospitalization, cardiovascular toxicity, and treatment-related mortality are of particular concern (12, 13). Older patients are especially vulnerable to these adverse outcomes because of age-related declines in bone marrow reserve, impaired organ function, comorbidity burden, and poor nutritional status (14). Recent evidence further suggests that febrile neutropenia often occurs early during treatment and reflects the clustering of multiple clinical risk factors rather than a single isolated event. In this context, primary prophylaxis with granulocyte colony-stimulating factor has been associated with lower febrile neutropenia risk and better treatment delivery, underscoring the importance of supportive care in preserving effective dose intensity (15).

Although current treatment strategies have improved the management of elderly DLBCL, important gaps remain in risk assessment and treatment individualization. Conventional prognostic tools have largely emphasized tumor burden and disease biology, but have not sufficiently incorporated age-related determinants such as functional status, comorbidity, and nutritional condition (16). As a result, prognostic discrimination within the elderly population often remains inadequate. In routine practice, clinicians frequently adopt attenuated regimens or empirical dose-reduction strategies, including R-miniCHOP or reduced relative dose intensity in frail or very old patients, yet the relationship between reduced treatment delivery and survival outcomes has not been consistently quantified across real-world cohorts. In addition, the specific patterns of dose reduction, delay, and interruption are often insufficiently documented and rarely integrated into prognostic assessment (17). More recently, geriatric-oriented prognostic models such as the Geriatric Prognostic Index have shown that combining age-related indicators, including activities of daily living, Charlson Comorbidity Index, and albumin, with disease-related variables such as stage, lactate dehydrogenase, and Eastern Cooperative Oncology Group performance status can improve survival stratification in elderly patients with DLBCL treated with immunochemotherapy (18, 19). These findings support growing calls to expand prognostic evaluation beyond lymphoma-specific disease burden alone and to incorporate functional, comorbid, and nutritional dimensions that are particularly relevant in older adults (20).

Against this background, the present single-center retrospective cohort study was undertaken to evaluate the efficacy, toxicity, and prognostic factors associated with first-line R-CHOP-based treatment in elderly patients with DLBCL. Specifically, we sought to characterize treatment outcomes in routine practice, with particular attention to treatment delivery, tolerability, and survival. We also aimed to identify clinically actionable prognostic factors associated with overall survival and to explore whether integrating geriatric and treatment-delivery features could improve risk stratification beyond conventional disease-based assessment. By clarifying which patients may benefit from stronger supportive care to maintain treatment intensity and which patients may be more suitable for treatment modification, this study aimed to provide clinically relevant evidence for individualized management in elderly DLBCL.

## 2 Methods

### 2.1 Study design and setting

This was a single-center retrospective cohort study conducted at Shanxi Cancer Hospital, Affiliated Cancer Hospital of Shanxi Medical University, Chinese Academy of Medical Sciences Cancer Hospital Shanxi Branch, Taiyuan, China. The study was designed to evaluate the efficacy, treatment-related toxicity, and prognostic factors associated with first-line rituximab plus cyclophosphamide, doxorubicin, vincristine, and prednisone (R-CHOP)-based treatment in elderly patients with diffuse large B-cell lymphoma (DLBCL). The study period extended from January 2013 to December 2023. Eligible cases were identified retrospectively from routinely collected institutional clinical data. This manuscript was prepared in accordance with the Strengthening the Reporting of Observational Studies in Epidemiology (STROBE) recommendations for retrospective cohort studies.

### 2.2 Ethics statement

This study was reviewed and approved by the institutional ethics committee of Shanxi Cancer Hospital. Because of the retrospective nature of the study and the use of de-identified clinical data obtained during routine care, the requirement for written informed consent was waived in accordance with institutional policy and the Declaration of Helsinki.

### 2.3 Participant identification and eligibility criteria

Eligible patients were identified through the institutional pathology information system and cross-checked against the inpatient electronic medical record system. Patients were included if they met all of the following criteria: (1) newly diagnosed DLBCL with pathological confirmation at our institution; (2) age 60 years or older at diagnosis; (3) receipt of first-line R-CHOP-based immunochemotherapy at Shanxi Cancer Hospital; and (4) availability of sufficient baseline clinical data, treatment records, and follow-up information for evaluation of the study outcomes. Patients were excluded if they had a pathological diagnosis other than DLBCL, were younger than 60 years, did not receive first-line R-CHOP-based treatment, had received prior systemic anti-lymphoma therapy, had transformed lymphoma, had human immunodeficiency virus-associated lymphoma, or lacked essential data required for efficacy, toxicity, or survival evaluation.

**Figure 1:**
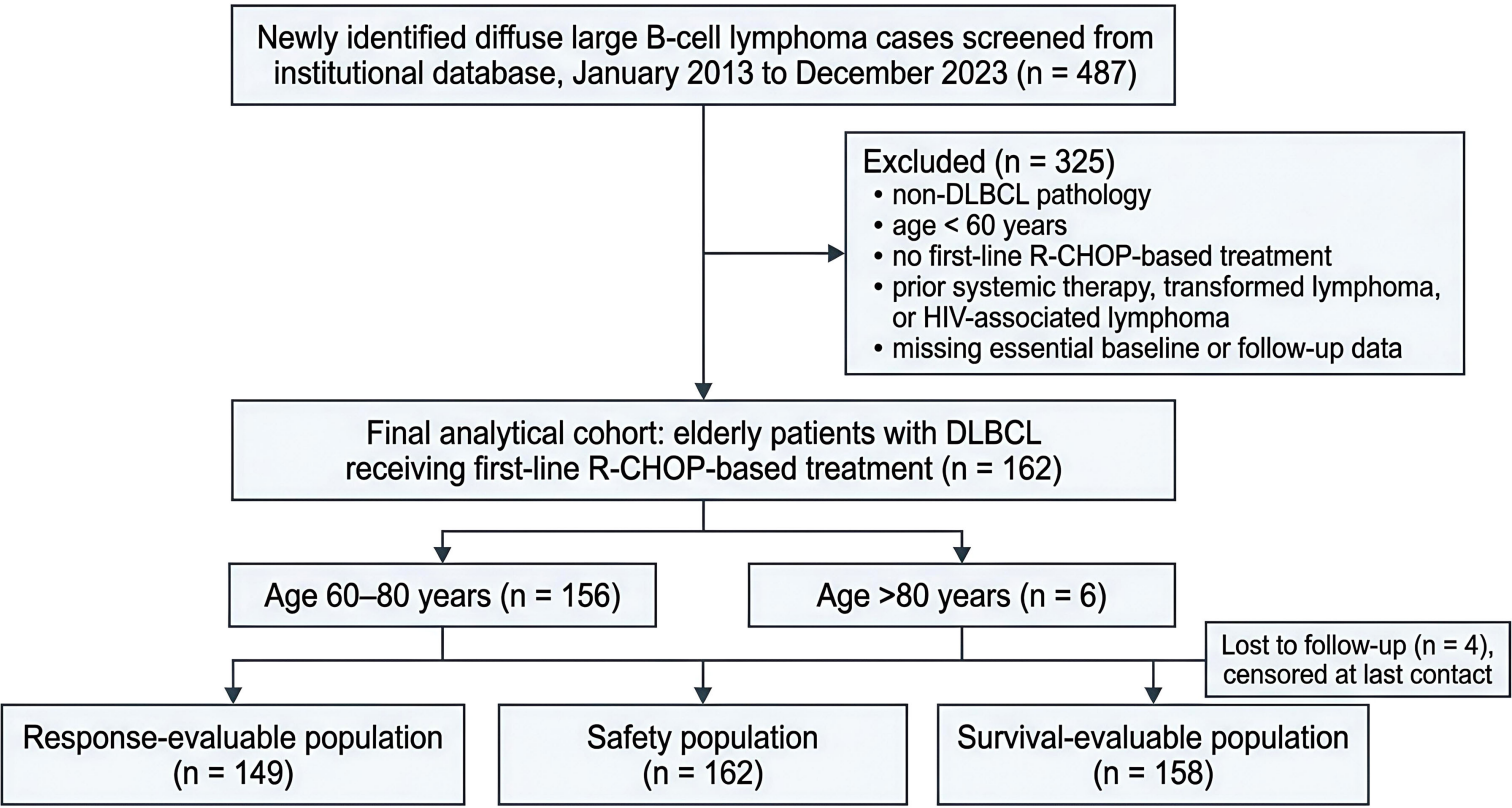
Flow diagram of patient selection and analysis populations. A total of 487 newly identified cases of diffuse large B-cell lymphoma were screened from the institutional database during the study period from January 2013 to December 2023. After application of the predefined inclusion and exclusion criteria, 325 records were excluded because of non-DLBCL pathology, age younger than 60 years, failure to receive first-line R-CHOP-based treatment, special clinical circumstances such as prior systemic therapy, transformed lymphoma, or human immunodeficiency virus-associated lymphoma, or missing essential baseline or follow-up data. The final analytical cohort consisted of 162 elderly patients aged 60 years or older. Among them, 149 patients were evaluable for response, 162 were included in the safety analysis, and 158 had follow-up data available for survival analysis. Four patients lost to follow-up were censored at the date of last contact according to the predefined rules.

A total of 487 records were initially retrieved from the institutional database. After application of the predefined inclusion and exclusion criteria, 325 records were excluded because of non-DLBCL diagnoses, age younger than 60 years, failure to receive first-line R-CHOP-based treatment, special clinical circumstances such as prior systemic treatment, transformed lymphoma, or human immunodeficiency virus-associated disease, or missing key baseline or follow-up data. The final analytical cohort consisted of 162 patients, including 156 patients aged 60–80 years and 6 patients aged older than 80 years. The detailed screening and enrollment process is shown in **Figure 1**.

### 2.4 Pathological diagnosis and baseline clinical assessment

All included cases were pathologically confirmed at the Department of Pathology of Shanxi Cancer Hospital. During cohort assembly, archived pathology reports and related histopathology records were independently reviewed by two senior hematopathologists to reconfirm the diagnosis of DLBCL. Pathological classification was based on the 2016 revision of the World Health Organization classification of lymphoid neoplasms.

Baseline clinical assessment was based on pretreatment medical records, laboratory results, and imaging findings. Disease stage was determined according to the Ann Arbor staging system. Extranodal involvement and bone marrow involvement were recorded from imaging studies, bone marrow examination results when available, and physician documentation in the medical record. Functional status was assessed using the Eastern Cooperative Oncology Group performance status scale. Baseline systemic symptoms, including B symptoms, were abstracted from physician notes recorded at diagnosis. These baseline domains were consistent with the variables prespecified in the original study framework, including stage, extranodal disease, performance status, hemoglobin, albumin, lactate dehydrogenase, and renal function.

### 2.5 Data sources and data abstraction

Clinical data were retrospectively extracted from multiple hospital information platforms to ensure comprehensive capture of baseline characteristics, treatment delivery, toxicity, and outcomes. The pathology information system was used to confirm diagnosis and classification. The electronic medical record system was used to obtain demographic characteristics, comorbidity history, performance status, presenting symptoms, disease characteristics, and physician-documented treatment plans. The chemotherapy prescription and administration system was used to extract cycle-specific treatment details, including planned doses, delivered doses, administration dates, dose adjustments, delays, and discontinuation. The laboratory information system was used to obtain baseline hematologic and biochemical variables, including complete blood count, lactate dehydrogenase, albumin, serum creatinine, and renal function-related data. The imaging reporting system was used to collect pretreatment staging information and post-treatment response assessments based on computed tomography or positron emission tomography/computed tomography. Follow-up information was obtained from hospital follow-up records, discharge summaries, and survival documentation available in the institutional system.

To reduce information bias, all study variables were extracted using a standardized case abstraction form developed before formal analysis. Logical consistency checks and source-to-source cross-validation were performed during data abstraction. Chemotherapy administration records were checked against hospitalization records and physician treatment notes. Serious adverse events were reviewed against laboratory abnormalities, inpatient course documentation, and discharge summaries. Survival outcomes were verified using follow-up records and related clinical documentation when available. This standardized extraction and verification process was consistent with the original study design, which prespecified key variable extraction and verification to improve transparency and reproducibility.

### 2.6 Treatment protocol and supportive care

All patients in the study cohort received first-line R-CHOP-based immunochemotherapy in routine clinical practice. The regimen consisted of rituximab combined with cyclophosphamide, doxorubicin, vincristine, and prednisone and was generally administered in 21-day cycles. The planned number of cycles and the final treatment intensity were determined by the treating hematology team according to patient age, disease burden, treatment response, organ function, performance status, comorbidity burden, and treatment tolerance.

Cycle-level treatment delivery variables were collected for each patient. These included treatment initiation date, number of administered cycles, planned and actual delivered doses per cycle, cycle interval, dose reduction, cycle delay, interruption, and premature discontinuation. Reasons for treatment modification or discontinuation were recorded when documented in the medical record and included toxicity, infection, disease progression, worsening comorbidities, poor general condition, organ dysfunction, and patient preference. Supportive care measures were recorded when available and included prophylactic or therapeutic granulocyte colony-stimulating factor, anti-infective treatment, blood transfusion, nutritional support, and other clinically indicated supportive interventions. Patients with pre-existing cardiovascular disease or elevated concern for anthracycline-related cardiotoxicity were monitored and managed according to institutional clinical practice during treatment. These supportive care domains were consistent with the original prespecified data framework, which explicitly included supportive therapy and treatment delivery information.

### 2.7 Baseline variables and prognostic indices

Baseline variables selected for analysis were chosen on the basis of clinical relevance in elderly DLBCL and availability in the institutional dataset. These variables included age, sex, Ann Arbor stage, B symptoms, extranodal involvement, bone marrow involvement, Eastern Cooperative Oncology Group performance status, hemoglobin, serum albumin, lactate dehydrogenase, and renal function. Comorbidity burden was assessed using documented medical history and medication records and was summarized using the Charlson Comorbidity Index when sufficient data were available.

The International Prognostic Index was calculated according to standard criteria when all required variables were available. The revised International Prognostic Index and National Comprehensive Cancer Network International Prognostic Index were additionally derived in patients with complete component data. These indices were used for descriptive stratification and for comparison with the study-specific geriatric-treatment delivery risk framework. This approach was consistent with the original study plan, which specified comparison with conventional prognostic systems including IPI and its modified versions.

### 2.8 Definitions of treatment delivery and dose intensity

Because treatment delivery is a central determinant of outcome in elderly patients with DLBCL, dose intensity-related variables were defined *a priori* and analyzed as major exposure variables. Cycle-by-cycle treatment data were extracted from chemotherapy prescribing and administration records, including planned and delivered doses, cycle dates, dose reductions, and delays. Dose reduction was defined as administration of a lower drug dose than originally planned for a given cycle. Cycle delay was defined as an unplanned postponement beyond the scheduled 21-day interval. Premature treatment discontinuation was defined as termination of planned treatment before completion of the intended course because of toxicity, disease progression, worsening comorbid conditions, or patient refusal. These definitions followed the original study concept that real-world treatment trajectories should be reconstructed through dose reduction, delay, interruption, and completion patterns.

Relative dose intensity was used to quantify treatment intensity and was calculated according to the standard method described by Hryniuk and Bush. Relative dose intensity was defined as the ratio of delivered dose intensity to the standard planned dose intensity, calculated according to the standard method described by Hryniuk and Bush (21). For the present study, relative dose intensity was derived from the major cytotoxic components of the R-CHOP regimen and analyzed both as a continuous variable and as a categorical variable using a prespecified threshold of 85% (≥85% vs.<85%). Treatment completion was additionally assessed according to completion of the planned treatment course or receipt of at least six cycles when this reflected the intended institutional treatment strategy. This threshold-based approach was aligned with the original draft, which specified stratified analysis using a cutoff of 85% to enhance clinical interpretability.

### 2.9 Outcome definitions

The primary study domains were efficacy, toxicity, and survival. Treatment response was assessed in patients with available post-treatment imaging and was classified according to the Lugano response criteria as complete response, partial response, stable disease, or progressive disease. Objective response rate was defined as the proportion of patients achieving complete or partial response, and complete response rate was analyzed separately. Patients without post-treatment imaging were excluded from efficacy analyses. In the present cohort, 149 patients were included in the response-evaluable population.

Treatment-related toxicity was graded according to the Common Terminology Criteria for Adverse Events, version 5.0. Safety analyses included all 162 patients who received first-line R-CHOP-based treatment. The safety analysis focused on clinically relevant adverse events in older patients, including grade 3 or higher hematologic toxicity, febrile neutropenia, hospitalization for severe infection, and treatment-related mortality. Treatment-related mortality was defined as death occurring within 90 days after initiation of first-line R-CHOP-based therapy that was attributable to treatment toxicity or treatment-associated complications rather than documented lymphoma progression, and this definition was applied consistently across all analyses. The original study draft had already prespecified febrile neutropenia, severe infection hospitalization, and treatment-related mortality as major safety endpoints.

Overall survival was defined as the time from initiation of first-line R-CHOP-based treatment to death from any cause. Progression-free survival was defined as the time from treatment initiation to disease progression, relapse, or death, whichever occurred first. Patients without an event were censored at the date of last follow-up. In the present cohort, survival analyses included 158 patients with follow-up data, and 4 patients lost to follow-up were censored at the date of last contact according to predefined follow-up rules. Follow-up duration was calculated in months. Your original draft had already specified the survival endpoints, follow-up censoring approach, and the existence of 4 lost-to-follow-up cases.

### 2.10 Bias control and study size

Several measures were used to reduce bias inherent to retrospective cohort studies. First, eligibility criteria were prespecified before data extraction. Second, all included cases were identified through institutional information systems rather than selective manual retrieval. Third, data extraction followed a standardized abstraction form with internal cross-checking to reduce misclassification and information bias. Fourth, outcome definitions, follow-up rules, and censoring principles were specified before statistical analysis. These procedures were consistent with the original study design, which explicitly stated that standardized variable extraction, outcome criteria, and follow-up rules had been defined at the design stage to improve comparability and rigor.

The study size was determined by the number of consecutive eligible patients treated at our center during the predefined study period. No formal sample size calculation was performed because of the retrospective design. The very small number of patients older than 80 years reflects the real-world distribution of eligible patients who received first-line R-CHOP-based treatment during the study period. Accordingly, analyses involving this subgroup were interpreted cautiously and primarily as exploratory age-stratified observations. This explanation is consistent with the original cohort composition of 156 patients aged 60–80 years and 6 patients aged older than 80 years.

### 2.11 Missing data

Variables were reviewed for completeness before analysis. Patients missing essential baseline or follow-up information required for evaluation of the primary study outcomes were excluded during cohort assembly. For the remaining cohort, analyses were performed on available data. Response analyses included only patients with post-treatment imaging, toxicity analyses included all 162 treated patients, and survival analyses included patients with follow-up information. Missingness for covariates used in multivariable modeling was handled by complete-case analysis. The original study draft already distinguished the response-evaluable population from the full cohort and specified censoring of lost-to-follow-up cases.

### 2.12 Statistical analysis

Continuous variables were summarized as mean ± standard deviation for normally distributed data and as median with interquartile range for non-normally distributed data. Categorical variables were presented as number and percentage. Between-group comparisons were performed using the independent-samples t test or Mann–Whitney U test for continuous variables, and the chi-square test or Fisher’s exact test for categorical variables, as appropriate. Overall survival and progression-free survival were estimated using the Kaplan–Meier method and compared using the log-rank test.

Cox proportional hazards regression was used to identify prognostic factors associated with overall survival. Variables considered clinically relevant or associated with outcome in univariable analysis were entered into multivariable models. To avoid model instability, closely related variables were not entered simultaneously when they reflected overlapping clinical constructs. Collinearity was assessed before multivariable modeling using correlation structure and variance inflation diagnostics. Hazard ratios and 95% confidence intervals were reported. Prespecified additional analyses were performed for treatment delivery variables, including relative dose intensity, treatment delay, dose reduction, and treatment completion, in order to examine the robustness of the association between treatment intensity and survival. The proportional hazards assumption was evaluated for Cox models using standard diagnostic methods based on time-dependent assessment and residual inspection. Discriminative performance of the study-specific geriatric-treatment delivery model and conventional prognostic indices was assessed using the concordance index. All statistical tests were two-sided, and P< 0.05 was considered statistically significant. Statistical analyses were performed using IBM SPSS Statistics for Windows, version 26.0 (IBM Corp., Armonk, NY, USA). The original study draft had already specified Kaplan–Meier analysis, Cox regression, and additional analyses around dose intensity-related variables.

## 3 Results

### 3.1 Patient selection and baseline cohort characteristics

During the study period from January 2013 to December 2023, 487 newly identified cases of diffuse large B-cell lymphoma were screened through the institutional database. After application of the predefined inclusion and exclusion criteria, 325 records were excluded because of non-DLBCL pathology, age younger than 60 years, failure to receive first-line R-CHOP-based treatment, special clinical circumstances such as prior systemic therapy, transformed lymphoma, or human immunodeficiency virus-associated lymphoma, or missing essential baseline or follow-up data. The final analytical cohort consisted of 162 elderly patients aged 60 years or older (**Figure 1**). Of these, 149 patients were evaluable for response, 162 were included in the safety analysis, and 158 had follow-up data available for survival analysis; 4 patients lost to follow-up were censored at the date of last contact according to the predefined rules.

The median age of the cohort was 72 years (range, 60–89), and 6 patients (3.7%) were older than 80 years. Baseline characteristics are summarized in **Table 1**. Overall, the cohort showed substantial disease burden and marked clinical heterogeneity: 97 patients (59.9%) had Ann Arbor stage III–IV disease, 91 (56.2%) had elevated lactate dehydrogenase, and 60 (37.0%) had a Charlson Comorbidity Index of 2 or higher. Compared with patients aged 60–80 years, those older than 80 years had poorer baseline functional reserve, including a higher proportion with ECOG performance status ≥2 (66.7% vs. 26.3%), Charlson Comorbidity Index ≥2 (83.3% vs. 35.3%), albumin<35 g/L (66.7% vs. 34.6%), and eGFR<60 mL/min/1.73 m^2^ (50.0% vs. 19.2%).

**Table 1:** Baseline clinical and laboratory characteristics of elderly patients with diffuse large B-cell lymphoma treated with R-CHOP.

Characteristics	Total cohort N=162	Age 60–80 years N=156	Age >80 years N=6
Demographic characteristics			
Age, years, median (range)	72 (60–89)	71 (60–80)	84 (81–89)
Male sex, n (%)	91 (56.2)	88 (56.4)	3 (50.0)
Clinical characteristics			
ECOG performance status ≥2, n (%)	45 (27.8)	41 (26.3)	4 (66.7)
Ann Arbor stage III–IV, n (%)	97 (59.9)	93 (59.6)	4 (66.7)
Bulky disease (≥7.5 cm), n (%)	29 (17.9)	28 (17.9)	1 (16.7)
Extranodal involvement >1 site, n (%)	37 (22.8)	36 (23.1)	1 (16.7)
Bone marrow involvement, n (%)	20 (12.3)	19 (12.2)	1 (16.7)
Charlson Comorbidity Index ≥2, n (%)	60 (37.0)	55 (35.3)	5 (83.3)
Laboratory characteristics			
Hemoglobin<100 g/L, n (%)	47 (29.0)	45 (28.8)	2 (33.3)
Albumin<35 g/L, n (%)	58 (35.8)	54 (34.6)	4 (66.7)
LDH >ULN, n (%)	91 (56.2)	88 (56.4)	3 (50.0)
eGFR<60 mL/min/1.73 m^2^, n (%)	33 (20.4)	30 (19.2)	3 (50.0)
Neutrophil-to-lymphocyte ratio ≥3, n (%)	78 (48.1)	74 (47.4)	4 (66.7)
IPI risk group, n (%)			
Low (0–1)	26 (16.0)	26 (16.7)	0 (0.0)
Low-intermediate (2)	45 (27.8)	44 (28.2)	1 (16.7)
High-intermediate (3)	56 (34.6)	54 (34.6)	2 (33.3)
High (4–5)	35 (21.6)	32 (20.5)	3 (50.0)
NCCN-IPI risk group, n (%)			
Low (0–1)	20 (12.3)	20 (12.8)	0 (0.0)
Low-intermediate (2–3)	71 (43.8)	70 (44.9)	1 (16.7)
High-intermediate (4–5)	50 (30.9)	48 (30.8)	2 (33.3)
High (≥6)	21 (13.0)	18 (11.5)	3 (50.0)

### 3.2 Treatment delivery, dose modifications, and supportive care

Treatment delivery data are summarized in **Table 2**. In the overall cohort, 124 of 162 patients (76.5%) completed at least 6 cycles of treatment. Treatment completion was substantially lower among patients older than 80 years, of whom only 2 of 6 patients (33.3%) completed at least 6 cycles, compared with 122 of 156 patients (78.2%) in the 60–80-year group (P = 0.024).

**Table 2:** Treatment delivery, dose modifications, and supportive care in the R-CHOP cohort.

Characteristics	Total cohort N=162	Age 60–80 years N=156	Age >80 years N=6	P Value
Treatment exposure				
Cycles of R-CHOP, median (range)	6 (1–8)	6 (1–8)	3.5 (1–6)	0.015
Patients completing ≥6 cycles, n (%)	124 (76.5)	122 (78.2)	2 (33.3)	0.024
Relative dose intensity (RDI)				
Average RDI (doxorubicin/cyclophosphamide), median (IQR)	92.5 (85.2–98.4)	93.6 (87.5–99.2)	78.2 (66.4–85.1)	0.009
RDI<85% (reduced intensity), n (%)	43 (26.5)	38 (24.4)	5 (83.3)	0.004
Dose modifications and delays				
Any dose reduction, n (%)	68 (42.0)	64 (41.0)	4 (66.7)	0.235
Due to hematologic toxicity, n (%)	38 (23.5)	35 (22.4)	3 (50.0)	0.138
Due to non-hematologic toxicity, n (%)	22 (13.6)	21 (13.5)	1 (16.7)	0.582
Treatment delay >7 days, n (%)	60 (37.0)	57 (36.5)	3 (50.0)	0.665
Early discontinuation<6 cycles, n (%)	38 (23.5)	34 (21.8)	4 (66.7)	0.024
Due to progression/death, n (%)	22 (13.6)	20 (12.8)	2 (33.3)	0.178
Due to toxicity/patient refusal, n (%)	16 (9.9)	14 (9.0)	2 (33.3)	0.115
Supportive care				
Anti-infective prophylaxis, n (%)	82 (50.6)	78 (50.0)	4 (66.7)	0.685
Hospitalization for chemotherapy administration, n (%)	142 (87.7)	136 (87.2)	6 (100.0)	1.000

^a^ P values compare patients aged 60–80 years with patients aged >80 years.

Relative dose intensity was significantly lower in the very elderly subgroup. The median relative dose intensity was 93.6% in patients aged 60–80 years and 78.2% in those older than 80 years (P = 0.009). Reduced-intensity treatment, defined as RDI<85%, was observed in 83.3% of patients older than 80 years. These findings indicate that treatment attenuation was concentrated in the oldest patients.

Dose modification remained common in routine practice. Patients older than 80 years showed higher rates of dose adjustment (66.7% vs. 41.0%) and treatment delay (50.0% vs. 36.5%) than those aged 60–80 years, although these differences did not reach statistical significance. Hematologic toxicity was the most frequent documented reason for unplanned dose reduction. Early discontinuation because of intolerance or patient refusal was also more common in the very elderly subgroup (33.3% vs. 9.0%). Supportive care was intensified in these patients according to clinical need.

### 3.3 Survival outcomes

**Figure 2:**
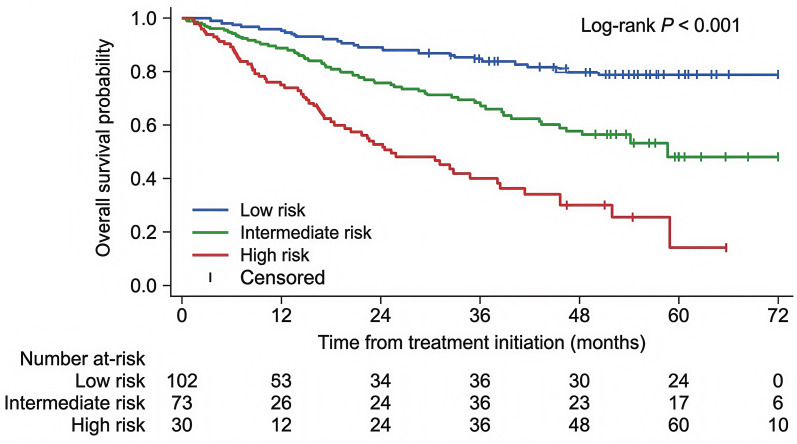
Kaplan–Meier curves for overall survival stratified by prespecified risk groups in elderly patients treated with R-CHOP. Kaplan–Meier analysis of overall survival according to the study-specific geriatric-RDI model. Patients were stratified into low-, intermediate-, and high-risk groups. The high-risk group showed significantly worse overall survival than the low- and intermediate-risk groups (log-rank P< 0.001).

**Figure 3:**
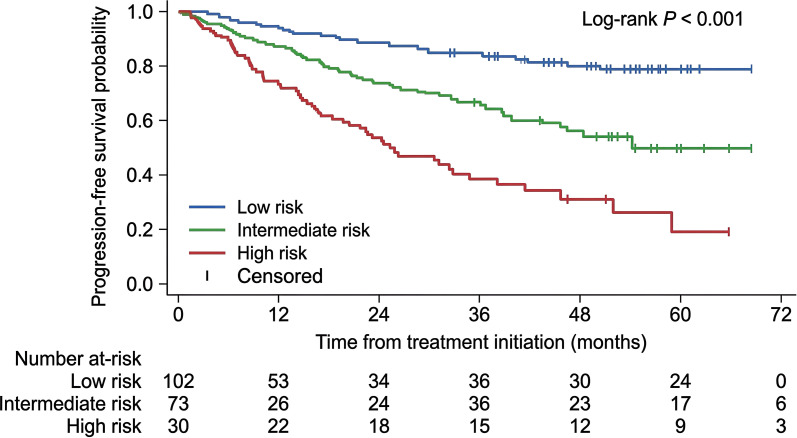
Kaplan–Meier curves for progression-free survival stratified by prespecified risk groups in elderly patients treated with R-CHOP. Kaplan–Meier analysis of progression-free survival according to the study-specific geriatric-RDI model. Patients in the high-risk group had the highest probability of progression or death (log-rank P< 0.001).

At a median follow-up of 28.4 months, median overall survival for the entire cohort had not been reached. Survival analyses were performed in 158 patients with follow-up data, with 4 lost-to-follow-up cases censored according to the predefined rules. Using the study-specific geriatric-RDI model, patients were stratified into low-, intermediate-, and high-risk groups. Kaplan–Meier analysis demonstrated clear separation of overall survival across these three strata (P< 0.001), with the high-risk group showing the poorest survival (**Figure 2**). The same model also discriminated progression-free survival, with the high-risk group showing the highest probability of progression or death (**Figure 3**).

**Figure 4:**
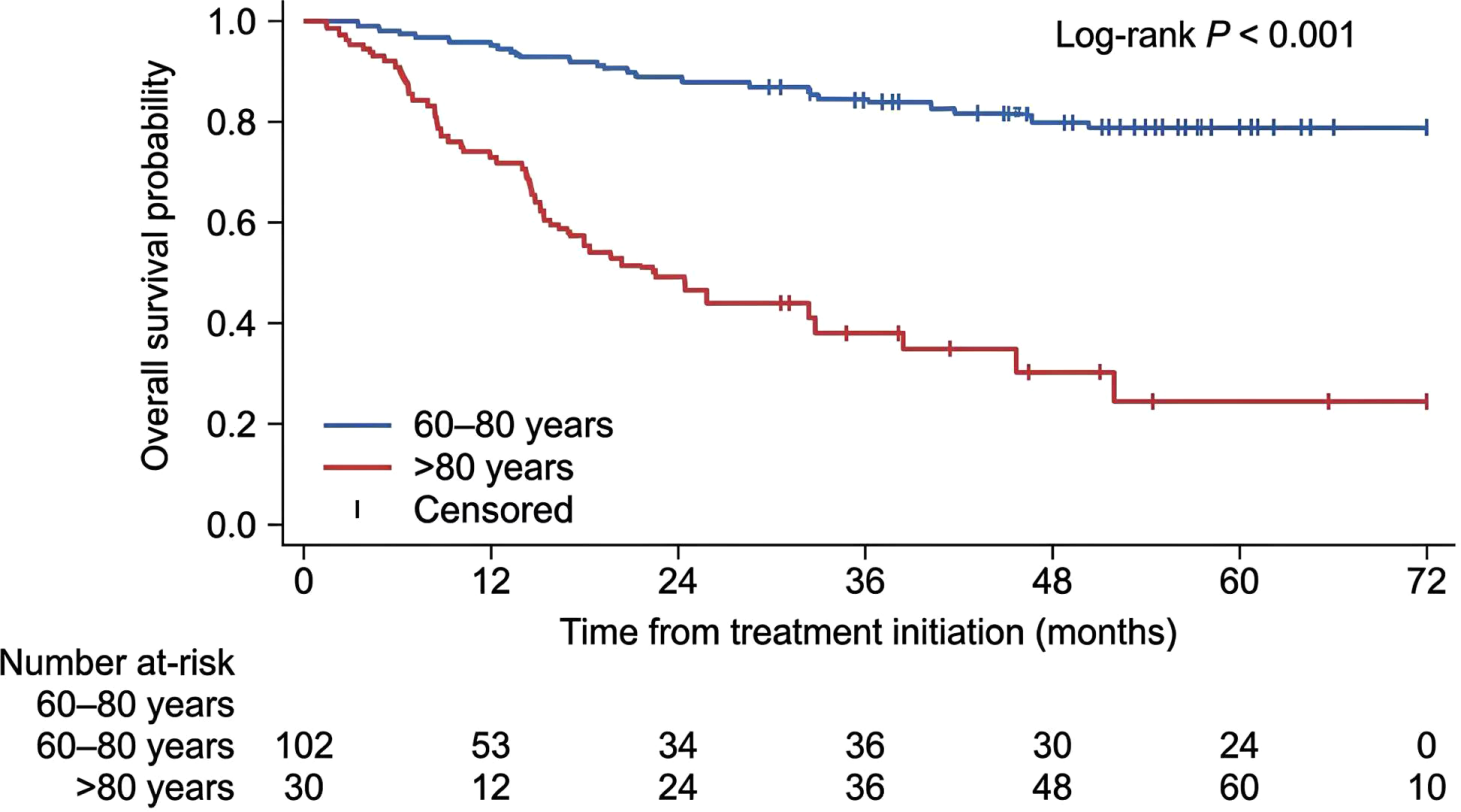
Overall survival stratified by age group (60–80 years vs >80 years). Kaplan–Meier curves showing overall survival according to age group. Patients older than 80 years had substantially inferior overall survival compared with patients aged 60–80 years (log-rank P< 0.001).

Age-stratified survival analysis further showed substantially inferior overall survival in patients older than 80 years compared with those aged 60–80 years (**Figure 4**, P< 0.001). Additional age-based subgroup displays suggested a stepwise decline in survival with increasing age, with the steepest separation observed in the oldest subgroup. These findings were consistent with the subsequent Cox regression analyses, in which advanced age remained an adverse prognostic factor.

### 3.4 Toxicity profile and treatment-related events

Major treatment-related toxicities are summarized in **Table 3**. Safety analyses included all 162 patients who received first-line R-CHOP-based treatment. Grade 3 or higher hematologic toxicity was common, with neutropenia representing the most frequent event, occurring in 101 patients (62.3%). Febrile neutropenia occurred in 25 patients (15.4%) overall, but the incidence was markedly higher in patients older than 80 years than in those aged 60–80 years (66.7% vs. 13.5%, P = 0.006).

**Table 3:** Major treatment-related toxicities and treatment-related events during R-CHOP.

Adverse Events (CTCAE Grade ≥3)^a^	Total cohort N=162	Age 60–80 years N=156	Age >80 years N=6	P Value^b^
Hematologic toxicities				
Neutropenia, n (%)	101 (62.3)	96 (61.5)	5 (83.3)	0.402
Febrile neutropenia, n (%)	25 (15.4)	21 (13.5)	4 (66.7)	0.006
Anemia, n (%)	36 (22.2)	34 (21.8)	2 (33.3)	0.605
Thrombocytopenia, n (%)	26 (16.0)	25 (16.0)	1 (16.7)	1.000
Non-hematologic toxicities				
Infection requiring hospitalization, n (%)	36 (22.2)	31 (19.9)	5 (83.3)	0.002
Pneumonia, n (%)	21 (13.0)	18 (11.5)	3 (50.0)	0.031
Sepsis/bacteremia, n (%)	8 (4.9)	6 (3.8)	2 (33.3)	0.038
Cardiac events, n (%)^c^	10 (6.2)	8 (5.1)	2 (33.3)	0.046
Heart failure (grade ≥3), n (%)	7 (4.3)	5 (3.2)	2 (33.3)	0.024
Arrhythmia (grade ≥3), n (%)	4 (2.5)	4 (2.6)	0 (0.0)	1.000
Gastrointestinal toxicity (mucositis/diarrhea), n (%)	12 (7.4)	11 (7.1)	1 (16.7)	0.405
Neuropathy (sensory/motor), n (%)	9 (5.6)	9 (5.8)	0 (0.0)	1.000
Fatigue/asthenia, n (%)	17 (10.5)	15 (9.6)	2 (33.3)	0.118
Treatment outcomes				
Treatment-related mortality, n (%)^d^	5 (3.1)	3 (1.9)	2 (33.3)	0.015

^a^ Toxicities were graded according to CTCAE version 5.0.

^b^ P values compare patients aged 60–80 years with patients aged >80 years.

^c^ Includes grade 3–4 cardiac adverse events.

^d^ Treatment-related mortality was defined as death occurring within 90 days after initiation of first-line R-CHOP-based therapy that was attributable to treatment toxicity or treatment-associated complications rather than documented lymphoma progression.

**Figure 5:**
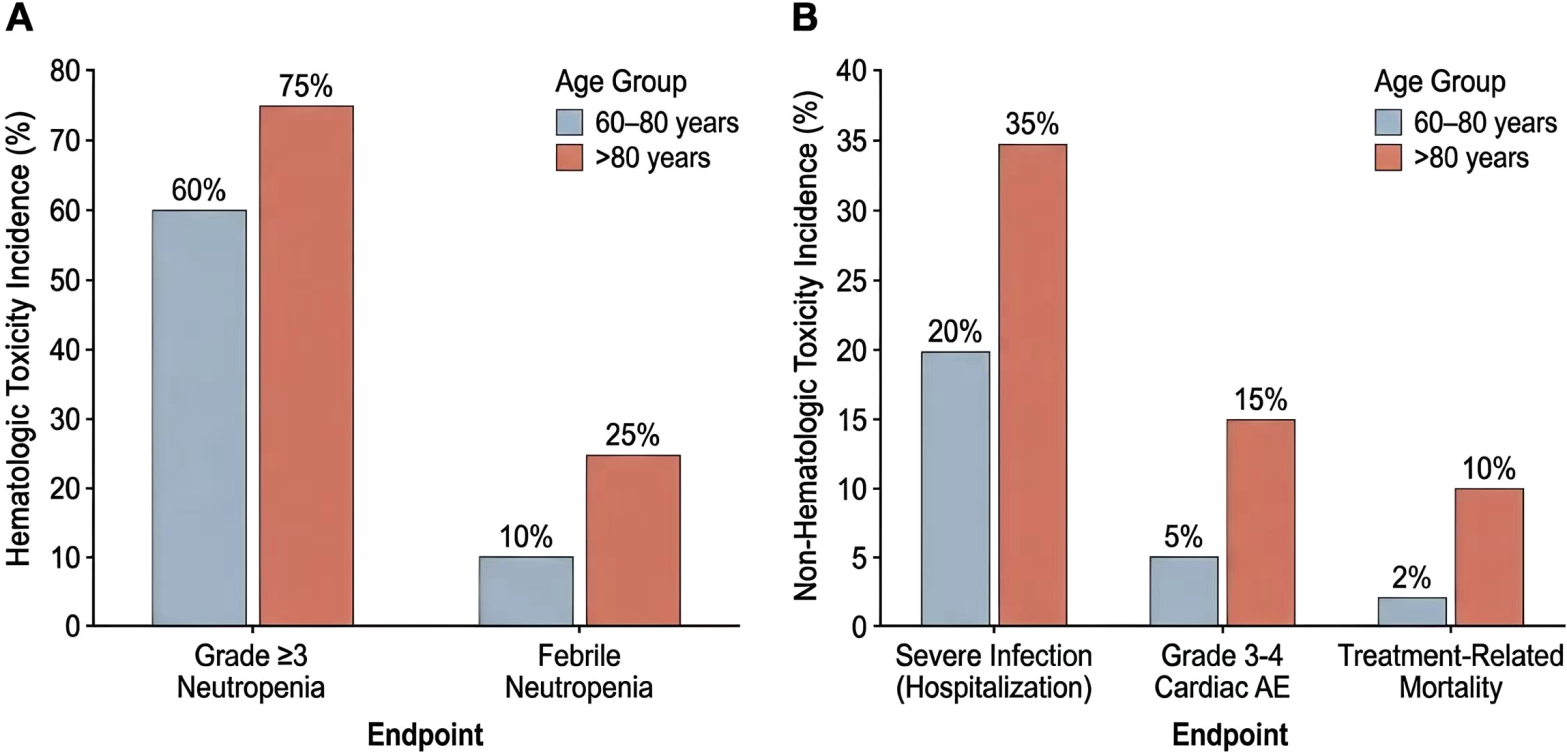
Major treatment-related toxicities in elderly patients receiving R-CHOP. **(A)** Age-stratified comparison of major hematologic toxicities, including grade ≥3 neutropenia and febrile neutropenia, in patients aged 60–80 years and >80 years. **(B)** Age-stratified comparison of major non-hematologic toxicities, including severe infection requiring hospitalization, grade 3–4 cardiac adverse events, and treatment-related mortality, in patients aged 60–80 years and >80 years.

Non-hematologic toxicity was likewise concentrated in the very elderly subgroup. Severe infection requiring hospitalization occurred in 36 patients (22.2%) overall and was substantially more common in patients older than 80 years than in the 60–80-year group (83.3% vs. 19.9%, P = 0.002). This included higher rates of pneumonia (50.0% vs. 11.5%, P = 0.031) and sepsis or bacteremia (33.3% vs. 3.8%, P = 0.038). Grade 3 or higher cardiac events were observed in 10 patients (6.2%) overall and again occurred more frequently in the >80-year group than in the 60–80-year group (33.3% vs. 5.1%, P = 0.046), mainly driven by severe heart failure (33.3% vs. 3.2%, P = 0.024). The distribution of the major hematologic and non-hematologic toxicities across age strata is shown in **Figures 5A, B**.

Treatment-related mortality also differed significantly by age stratum. Under the prespecified study definition, treatment-related mortality occurred in 5 patients (3.1%) overall, including 2 of 6 patients older than 80 years (33.3%) and 3 of 156 patients aged 60–80 years (1.9%; P = 0.015). These findings indicate a substantially narrower therapeutic window in the very elderly population. The age-stratified pattern of major treatment-related toxicities, including severe infection, cardiac events, and treatment-related mortality, is further illustrated in **Figure 5**.

**Figure 6:**
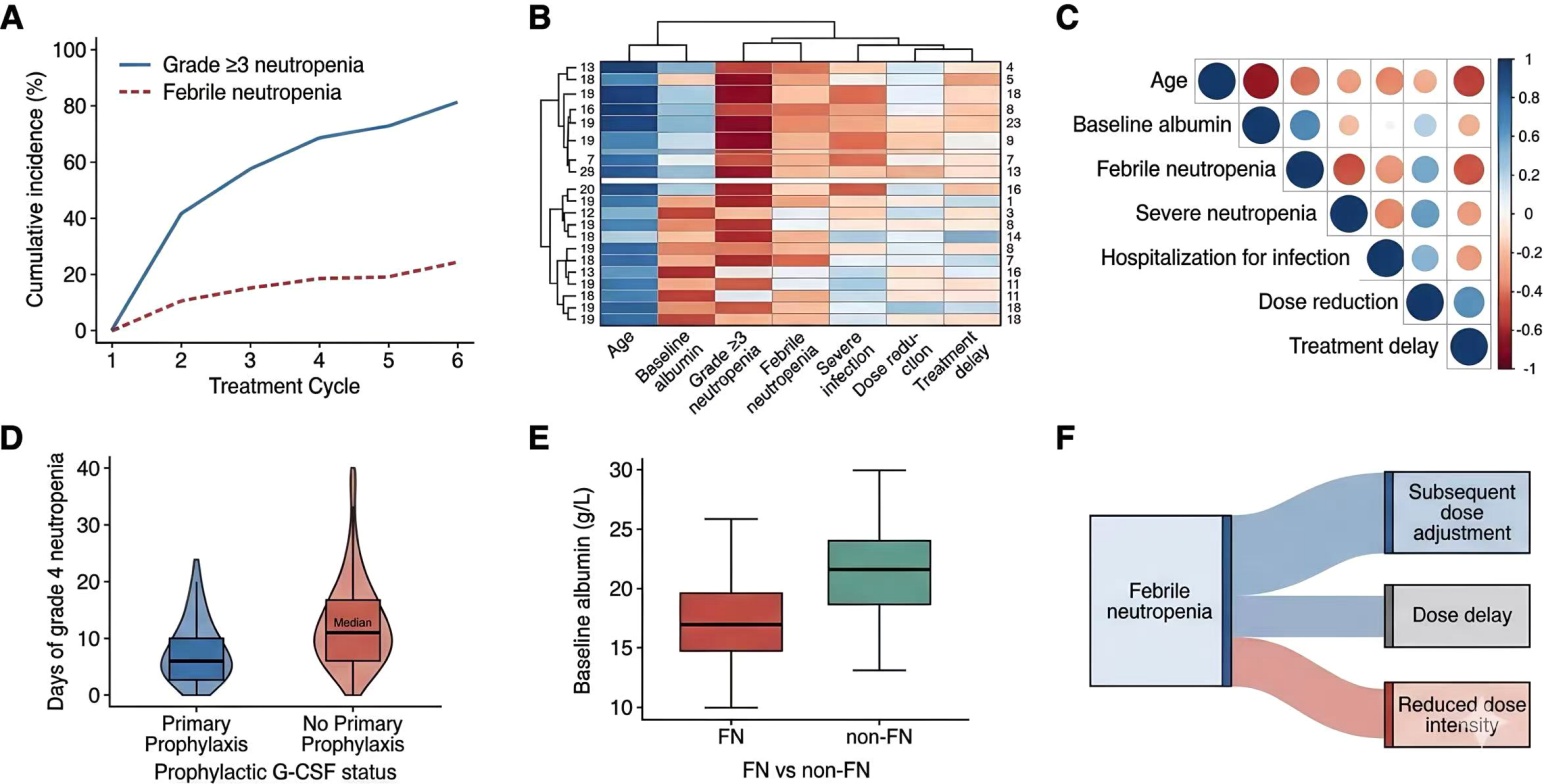
Exploratory analyses of hematologic toxicity dynamics and treatment attenuation during R-CHOP. **(A)** Cumulative incidence of grade ≥3 neutropenia and febrile neutropenia across treatment cycles. **(B)** Patient-level clustered heatmap illustrating toxicity burden patterns across elderly patients. **(C)** Correlation matrix showing the relationships among age, baseline albumin, febrile neutropenia, severe neutropenia, infection-related hospitalization, dose reduction, and treatment delay. **(D)** Comparison of duration of grade 4 neutropenia according to use of primary prophylactic granulocyte colony-stimulating factor. **(E)** Distribution of baseline albumin according to febrile neutropenia status. **(F)** Treatment trajectory analysis showing the association between febrile neutropenia and subsequent dose adjustment, delay, or treatment attenuation.

Figure-based exploratory analyses further suggested that severe hematologic events clustered early during treatment and accumulated over successive cycles (**Figure 6A**). Additional exploratory displays indicated that older age and lower baseline albumin were associated with a higher toxicity burden (**Figures 6B, C**). Patients who developed febrile neutropenia had lower baseline albumin levels than those who did not (**Figure 6E**). In descriptive comparisons, primary prophylactic granulocyte colony-stimulating factor was associated with a shorter duration of grade 4 neutropenia (**Figure 6D**). Febrile neutropenia was also followed by a higher probability of subsequent dose adjustment, supporting the clinical link between early toxicity and later treatment attenuation (**Figure 6F**).

### 3.5 Prognostic factors for overall survival

Univariable Cox regression analyses are presented in **Table 4**. Older age (≥80 vs.<80 years; HR, 2.45; 95% CI, 1.56–3.84; P< 0.001), ECOG performance status ≥2 (HR, 2.15; 95% CI, 1.38–3.35; P = 0.001), Charlson Comorbidity Index ≥2 (HR, 1.84; 95% CI, 1.16–2.92; P = 0.009), Ann Arbor stage III–IV (HR, 1.95; 95% CI, 1.18–3.22; P = 0.009), elevated lactate dehydrogenase, hypoalbuminemia, and reduced relative dose intensity were all associated with inferior overall survival. In particular, albumin<35 g/L (HR, 2.08; P = 0.002) and RDI<85% (HR, 2.68; 95% CI, 1.65–4.35; P< 0.001) showed strong adverse associations with survival.

**Table 4:** Univariable Cox regression analyses of candidate predictors for overall survival.

Variable	Hazard ratio (HR)	95% CI	P Value
Demographic and clinical variables			
Age (≥80 vs<80 years)	2.45	1.56–3.84	<0.001
Sex (male vs female)	1.12	0.71–1.76	0.624
ECOG performance status (≥2 vs 0–1)	2.15	1.38–3.35	0.001
Charlson Comorbidity Index (≥2 vs<2)	1.84	1.16–2.92	0.009
Tumor characteristics			
Ann Arbor stage (III–IV vs I–II)	1.95	1.18–3.22	0.009
Bulky disease (≥7.5 cm vs absent)	1.34	0.78–2.31	0.287
Extranodal sites (>1 vs ≤1)	1.62	1.01–2.59	0.045
Bone marrow involvement (yes vs no)	1.58	0.94–2.66	0.082
Laboratory variables			
LDH (>ULN vs normal)	2.23	1.39–3.58	0.001
Albumin (<35 vs ≥35 g/L)	2.08	1.32–3.28	0.002
Hemoglobin (<100 vs ≥100 g/L)	1.65	1.05–2.59	0.031
eGFR (<60 vs ≥60 mL/min/1.73 m^2^)	1.48	0.91–2.41	0.113
Treatment variable			
Relative dose intensity (<85% vs ≥85%)	2.68	1.65–4.35	<0.001

**Figure 7:**
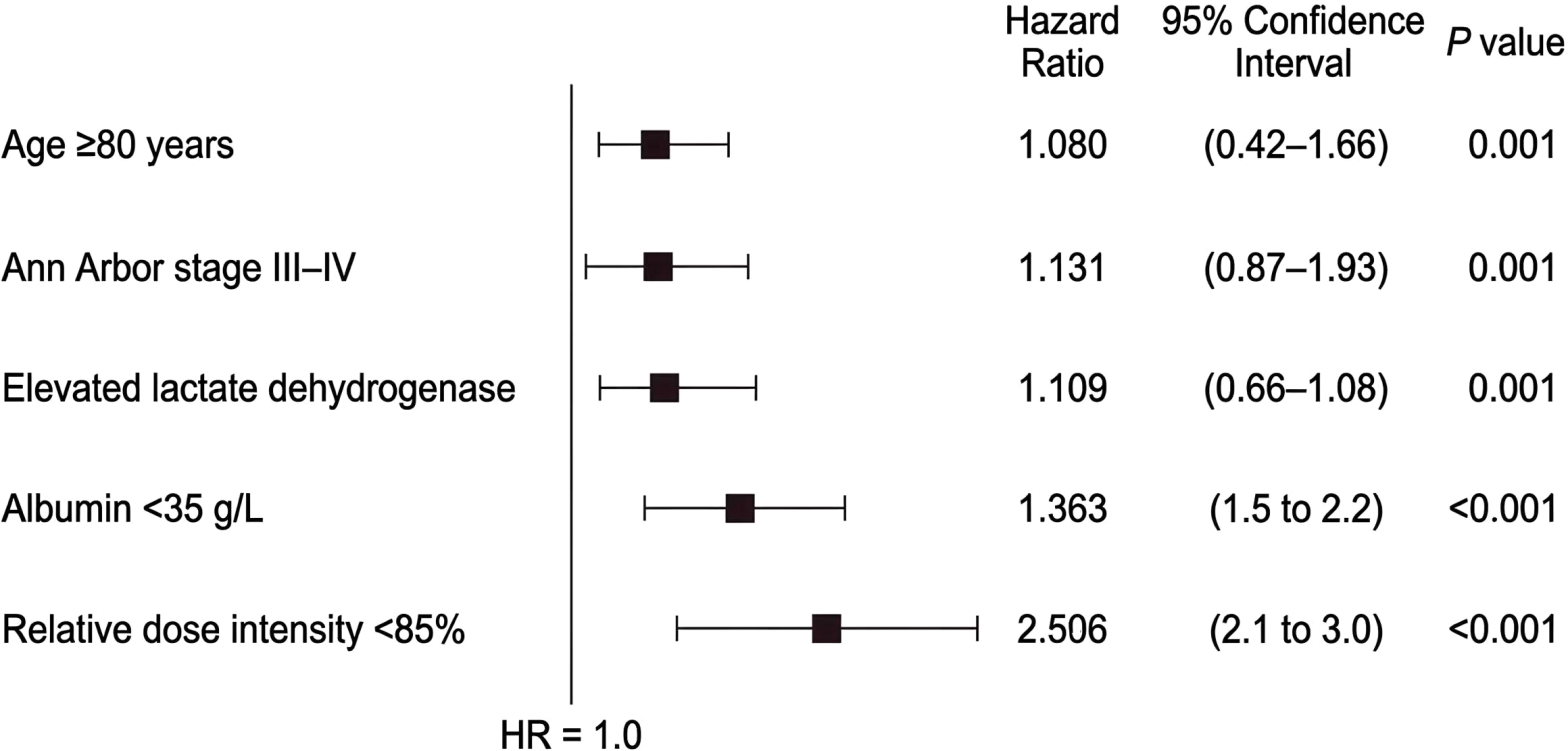
Forest plot of the multivariable Cox regression model for overall survival. Forest plot showing hazard ratios and 95% confidence intervals for the independent prognostic factors retained in the multivariable Cox model, including age ≥80 years, Ann Arbor stage III–IV, elevated lactate dehydrogenase, albumin<35 g/L, and relative dose intensity<85%.

When candidate variables were entered into the multivariable Cox model, age, disease burden, nutritional status, and treatment intensity remained the principal determinants of outcome (**Table 5**). Specifically, age ≥80 years, Ann Arbor stage III–IV, elevated lactate dehydrogenase, albumin<35 g/L, and RDI<85% remained independently associated with worse overall survival. Among these variables, reduced relative dose intensity remained the strongest independent predictor of poor prognosis (HR, 2.45; 95% CI, 1.65–3.63; P< 0.001), while hypoalbuminemia also retained independent prognostic significance (HR, 2.03; 95% CI, 1.38–2.99; P< 0.001). By contrast, ECOG performance status and Charlson Comorbidity Index were attenuated after adjustment and were no longer independently significant, consistent with overlap between baseline frailty, age, and treatment tolerance. The relative effects of the retained covariates are illustrated in **Figure 7**.

**Table 5:** Multivariable Cox regression model for overall survival in elderly patients receiving R-CHOP.

Variable	Hazard ratio (HR)	95% CI	P Value
Age (≥80 vs<80 years)	1.92	1.25–2.95	0.003
Ann Arbor stage (III–IV vs I–II)	1.62	1.09–2.41	0.017
LDH (>ULN vs normal)	1.51	1.03–2.22	0.035
Albumin (<35 vs ≥35 g/L)	2.03	1.38–2.99	<0.001
Relative dose intensity (<85% vs ≥85%)	2.45	1.65–3.63	<0.001
ECOG performance status (≥2 vs 0–1)^a^	1.38	0.85–2.24	0.192
Charlson Comorbidity Index (≥2 vs<2)^a^	1.25	0.76–2.06	0.385

^a^ Included in the multivariable model but not independently significant after adjustment.

### 3.6 Model extension, sensitivity analyses, and score comparison

To further assess the robustness of the main findings, we performed prespecified model extension and sensitivity analyses using alternative adjustment strategies (**Table 6**). In the extended model that additionally adjusted for prophylactic granulocyte colony-stimulating factor use and Charlson Comorbidity Index, reduced treatment intensity remained significantly associated with inferior overall survival, with RDI<85% continuing to predict poor prognosis (HR, 1.98; P = 0.012). This finding suggests that the association between reduced dose intensity and survival was not explained solely by differences in comorbidity burden or supportive care strategy.

**Table 6:** Sensitivity and extended Cox models for overall survival incorporating dose intensity and treatment delivery.

Model scenarios and variables	Hazard ratio (HR)	95% CI	P value
Model A: Extended adjustment model^a^			
Age ≥80 years	1.76	1.05–2.95	0.032
Albumin<35 g/L	1.62	1.03–2.54	0.036
Relative dose intensity<85%	1.98	1.16–3.39	0.012
Charlson Comorbidity Index ≥2	1.15	0.68–1.94	0.605
G-CSF use (primary/secondary)	0.88	0.54–1.42	0.598
Model B: 3-month landmark analysis^b^			
Age ≥80 years	1.92	1.14–3.23	0.014
Ann Arbor stage III–IV	1.58	0.98–2.55	0.061
Relative dose intensity<85%	1.85	1.09–3.14	0.023
Treatment completion (≥6 cycles)	0.65	0.38–1.12	0.121
Model C: Exploratory subgroup analysis in patients aged >80 years^c^			
Albumin<35 g/L	2.45	1.12–5.36	0.025
Relative dose intensity<85%	3.12	1.45–6.71	0.004
ECOG performance status ≥2	1.54	0.72–3.29	0.264

^a^ Extended adjustment model additionally included Charlson Comorbidity Index and granulocyte colony-stimulating factor use alongside the core predictors.

^b^ Landmark analysis excluded patients who died or were lost to follow-up within 3 months after treatment initiation.

^c^ Restricted to the oldest subgroup (N = 6) and should be interpreted as exploratory because of the very small sample size.

To reduce the potential influence of early deaths and reverse causality on treatment-intensity estimates, we next performed a 3-month landmark analysis. Patients who died or were lost to follow-up within the first 3 months after treatment initiation were excluded from this analysis. In this landmark model, RDI<85% remained significantly associated with worse survival (HR, 1.85; P = 0.023), supporting the stability of the main result.

We also performed an exploratory model restricted to patients older than 80 years (Model C). Within this very small subgroup, both hypoalbuminemia and reduced RDI continued to show large adverse effect estimates (albumin<35 g/L: HR, 2.45; RDI<85%: HR, 3.12). However, because this subgroup analysis was based on only 6 patients, these results should be interpreted cautiously as exploratory observations rather than definitive estimates.

The geriatric-RDI model, which was derived from the independent prognostic factors identified above, was then compared with existing clinical scoring systems (**Table 7**). This model showed superior discrimination for overall survival, with a Harrell’s C-index of 0.748 (95% CI, 0.691–0.805), compared with 0.635 for the International Prognostic Index, 0.641 for the revised International Prognostic Index, and 0.682 for the National Comprehensive Cancer Network International Prognostic Index. In addition, the geriatric-RDI model yielded the lowest Akaike information criterion value (798.4), representing a 46.8-point reduction relative to the International Prognostic Index model, indicating improved model fit for survival prediction in elderly patients with diffuse large B-cell lymphoma.

**Table 7:** Internal validation and comparative performance of prognostic scores for overall survival.

Prognostic score/Model	Harrell’s C-Index (95% CI)	Bootstrap C-Index^a^	AIC	ΔAIC vs IPI
Standard clinical scores				
IPI (International Prognostic Index)	0.635 (0.572–0.698)	0.628	845.2	Ref.
R-IPI (Revised IPI)	0.641 (0.580–0.702)	0.636	841.8	-3.4
NCCN-IPI	0.682 (0.621–0.743)	0.675	829.5	-15.7
Study-specific model				
Geriatric-RDI model^b^	0.748 (0.691–0.805)	0.739	798.4	-46.8

^a^ Bootstrap internal validation based on 1,000 resamples.

^b^ Study-specific geriatric-RDI model included age, stage, LDH, albumin, and relative dose intensity.

Internal validation using 1,000 bootstrap resamples produced a corrected C-index of 0.739, suggesting only limited optimism and no evidence of substantial overfitting. Taken together, these findings support the incremental prognostic value of incorporating both geriatric reserve and treatment-delivery variables into risk assessment.

### 3.7 Subgroup analyses in very old patients and by prespecified clinical strata

We further explored treatment allocation patterns within the subgroup of patients older than 80 years (n = 6) (**Table 8**). In this very old subgroup, patients who maintained RDI ≥85% appeared to have more favorable baseline physiological reserve than those treated at reduced intensity. Specifically, the higher-RDI group had a lower median age (81 vs. 88 years; P = 0.046) and a lower proportion of patients with poor performance status (ECOG ≥2: 33.3% vs. 100.0%; P = 0.400). Hypoalbuminemia was also more frequent among patients treated at reduced intensity (100.0% vs. 33.3%; P = 0.400). By contrast, tumor-burden indicators such as Ann Arbor stage, lactate dehydrogenase level, and International Prognostic Index risk category showed no clear between-group difference within this subgroup. Although these comparisons were limited by the very small sample size, the overall pattern suggests that dose attenuation in the oldest patients was more strongly related to frailty and reduced physiological reserve than to lymphoma burden alone.

**Table 8:** Baseline characteristics of patients aged >80 years stratified by relative dose intensity.

Characteristics	Total N=6	RDI ≥85% N=3	RDI<85% N=3	P value
Demographic characteristics				
Age, years, median (range)	84 (81–89)	81 (81–82)	88 (86–89)	0.046
Male sex, n (%)	3 (50.0)	1 (33.3)	2 (66.7)	1.000
Functional and comorbidity status				
ECOG performance status ≥2, n (%)	4 (66.7)	1 (33.3)	3 (100.0)	0.400
Charlson Comorbidity Index ≥2, n (%)	5 (83.3)	2 (66.7)	3 (100.0)	1.000
ADL dependence, n (%)	2 (33.3)	0 (0.0)	2 (66.7)	0.400
Tumor burden and disease risk				
Ann Arbor stage III–IV, n (%)	4 (66.7)	2 (66.7)	2 (66.7)	1.000
Bulky disease (≥7.5 cm), n (%)	1 (16.7)	0 (0.0)	1 (33.3)	1.000
LDH >ULN, n (%)	3 (50.0)	1 (33.3)	2 (66.7)	1.000
Physiologic reserve				
Albumin<35 g/L, n (%)	4 (66.7)	1 (33.3)	3 (100.0)	0.400
eGFR<60 mL/min/1.73 m^2^, n (%)	3 (50.0)	1 (33.3)	2 (66.7)	1.000
Treatment planning				
Planned 6 cycles, n (%)	3 (50.0)	3 (100.0)	0 (0.0)	0.100
Planned<6 cycles (attenuated-intensity strategy), n (%)	3 (50.0)	0 (0.0)	3 (100.0)	0.100

^a^ Because of the extremely small sample size, all comparisons in this table should be interpreted as exploratory and descriptive.

**Figure 8:**
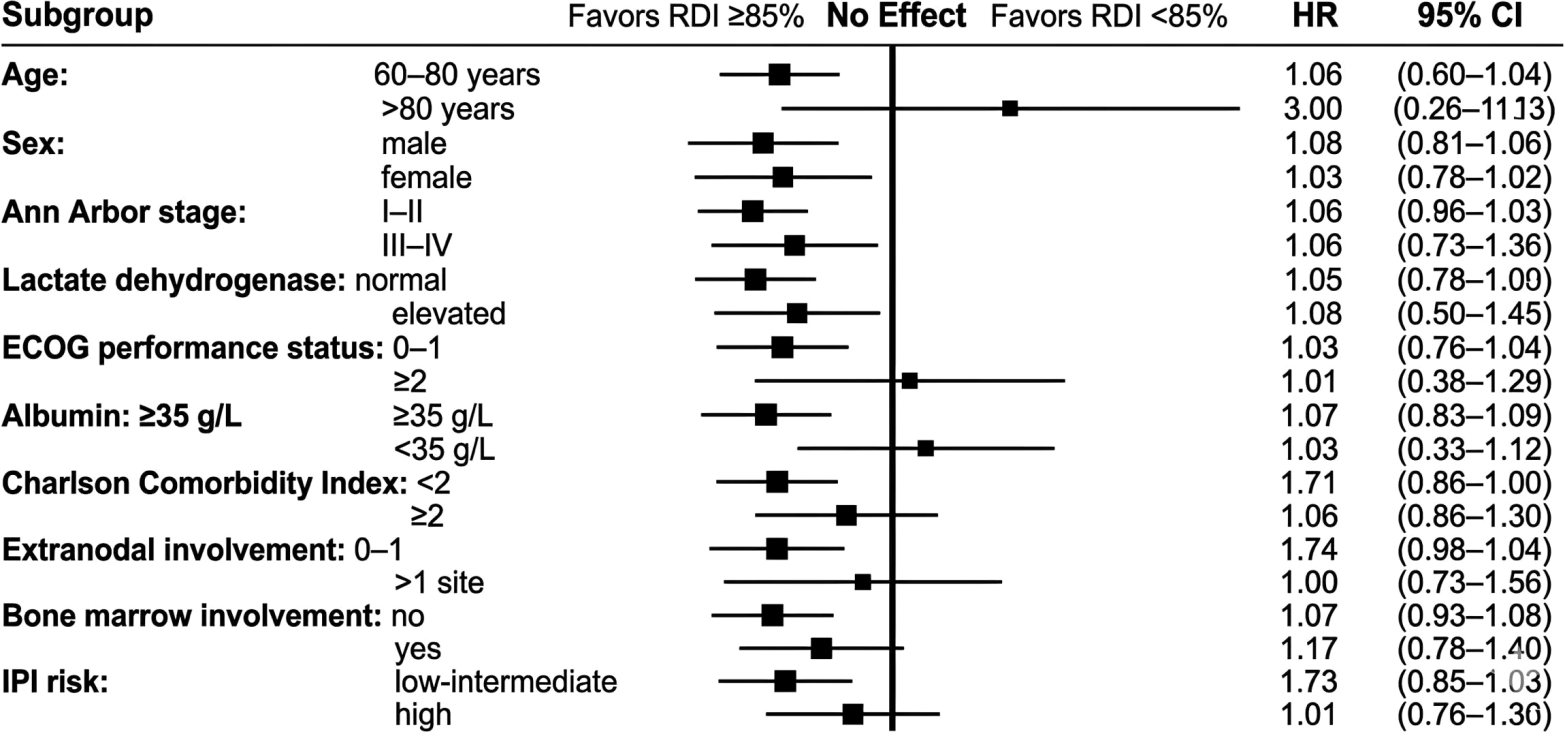
Subgroup analyses of overall survival according to prespecified clinical factors and dose intensity. Forest plot of subgroup analyses comparing the prognostic effect of relative dose intensity ≥85% versus<85% across prespecified clinical strata, including age, sex, stage, lactate dehydrogenase, ECOG performance status, albumin, Charlson Comorbidity Index, extranodal involvement, bone marrow involvement, and International Prognostic Index risk category.

To examine whether the prognostic effect of treatment intensity was consistent across different clinical strata, we also performed subgroup survival analyses (**Figure 8**). The survival advantage associated with RDI ≥85% was generally preserved across most prespecified subgroups, with more apparent benefit in patients with lower lactate dehydrogenase levels and localized-stage disease. In contrast, among patients with ECOG performance status ≥2 or marked hypoalbuminemia, the confidence intervals for the effect of RDI crossed the null line. These findings suggest that the survival benefit associated with maintaining standard dose intensity may be attenuated in patients with severely impaired physiological reserve.

Because these subgroup analyses were exploratory, they should be interpreted cautiously. Nonetheless, they provide clinically relevant context for the main findings by suggesting that the relationship between treatment intensity and survival is not uniform across all elderly patients, and may depend in part on the patient’s underlying functional and nutritional status.

## 4 Discussion

In this single-center retrospective cohort of elderly patients aged 60 years or older with diffuse large B-cell lymphoma who received first-line R-CHOP-based treatment, we found that survival outcomes were shaped not only by lymphoma burden, but also by treatment delivery and patient-level vulnerability (22, 23). In addition to established disease-related factors such as advanced stage and elevated lactate dehydrogenase, age, nutritional status, and relative dose intensity contributed substantially to prognosis. In the multivariable model, reduced treatment intensity (RDI<85%) and hypoalbuminemia (albumin<35 g/L) remained independently associated with inferior overall survival, indicating that prognosis in older patients is influenced by both tumor biology and the ability to tolerate and sustain curative-intent therapy.

These findings are clinically important because they extend risk assessment beyond traditional lymphoma-centered scoring systems. Conventional tools such as the International Prognostic Index remain useful for baseline disease stratification, but they do not fully capture the heterogeneity of physiological reserve in older adults. Our results are consistent with the broader geriatric lymphoma literature showing that functional status, comorbidity burden, and nutritional condition materially affect treatment tolerance and long-term outcome. In this context, the better performance of the geriatric-RDI model compared with IPI-based systems suggests that integrating treatment-delivery variables with geriatric vulnerability features may provide a more clinically relevant approach to risk assessment in elderly patients receiving immunochemotherapy.

A central finding of the present study is the strong and consistent association between reduced dose intensity and worse survival. In routine practice, maintenance of standard R-CHOP intensity in older patients is frequently compromised by hematologic toxicity, infection, frailty, organ dysfunction, and clinician concern regarding tolerability. However, our results suggest that reduced treatment delivery is not merely a marker of treatment modification, but a clinically meaningful determinant of outcome. Even after adjustment for age, disease burden, and other baseline covariates, RDI<85% remained the strongest independent adverse prognostic factor. This pattern was preserved in the extended model and in the landmark analysis, supporting the robustness of the association.

These observations align with current clinical thinking in elderly diffuse large B-cell lymphoma. For patients with adequate physiological reserve, curative-intent immunochemotherapy should be delivered as effectively as possible rather than being routinely attenuated on the basis of chronological age alone (24, 25). By contrast, for patients who are clearly frail or unfit, the question is not simply whether to reduce dose intensity, but how to individualize treatment in a way that avoids both undertreatment and excessive toxicity (26–28). Our results support a suitability-based rather than age-based treatment strategy. In other words, dose reduction should not be adopted as a blanket response to older age; instead, it should follow structured assessment of functional reserve, comorbidity burden, nutritional status, and anticipated treatment tolerance (29).

The subgroup findings in patients older than 80 years, although exploratory because of the very small sample size, point in the same direction. Patients who maintained RDI ≥85% appeared to have better baseline physiological reserve than those treated at reduced intensity, whereas tumor-burden indicators showed less obvious separation. This pattern suggests that in very old patients, treatment attenuation may be driven more by frailty and reduced reserve than by lymphoma aggressiveness itself. The clinical implication is not that all very old patients should receive standard-intensity therapy, but rather that treatment intensity should be individualized on the basis of vulnerability assessment rather than age category alone.

Toxicity findings from this study provide an important explanation for why treatment intensity is difficult to maintain in older patients. Grade 3 or higher hematologic toxicity was common across the cohort, and febrile neutropenia, severe infection requiring hospitalization, cardiac events, and treatment-related mortality were all markedly more frequent in patients older than 80 years. These results indicate that toxicity is not a peripheral issue in elderly R-CHOP delivery; it is one of the main pathways through which prognosis worsens. Once major toxicity occurs, treatment is more likely to be delayed, attenuated, or discontinued, thereby weakening the continuity and curative potential of frontline therapy.

This is particularly evident in the relationship among age, albumin, toxicity, and treatment delivery. The exploratory analyses suggested that older age and lower baseline albumin were associated with greater toxicity burden, and patients who developed febrile neutropenia tended to have lower baseline albumin levels. Although these analyses were descriptive, they reinforce a clinically plausible interpretation: reduced nutritional reserve may limit hematologic resilience and increase vulnerability to treatment interruption. In this setting, supportive care should not be viewed as secondary to anti-lymphoma treatment. Rather, it should be considered an essential part of curative-intent management, especially in older patients at increased risk of febrile neutropenia, infection, or early treatment attenuation (30, 31).

The practical implications of these findings are clear. Before treatment initiation, clinicians should not rely solely on age or lymphoma stage when planning therapy. Instead, baseline evaluation should incorporate functional status, comorbidity burden, albumin level, and other markers of physiological reserve. During treatment, patients at elevated risk of toxicity may benefit from closer infection surveillance, earlier supportive intervention, nutritional optimization, and individualized prevention strategies such as appropriate granulocyte colony-stimulating factor use (15, 30, 32, 33). The purpose of these measures is not simply to reduce toxicity in isolation, but to preserve treatment continuity and sustain adequate dose intensity in patients with curative potential.

This study also has several limitations. First, it was a single-center retrospective analysis and is therefore subject to the usual risks of selection bias, residual confounding, and incomplete documentation. Second, the subgroup of patients older than 80 years was very small, which limits the precision of age-stratified estimates and requires cautious interpretation of subgroup findings. Third, although the geriatric-RDI model showed better discrimination than conventional scoring systems, it remains a study-specific model and requires external validation before broader clinical adoption. Finally, treatment allocation and dose modification were based on real-world physician decision-making rather than protocolized intervention, which reflects routine practice but may also introduce confounding by indication.

Despite these limitations, this study provides a clinically actionable perspective on frontline treatment in elderly diffuse large B-cell lymphoma. The findings suggest that optimal management requires integration of three elements: vulnerability stratification before treatment, maintenance of effective dose intensity whenever feasible, and proactive toxicity prevention throughout therapy. Future prospective studies should evaluate whether simplified geriatric assessment tools, combined with dynamic treatment-delivery monitoring and structured supportive care pathways, can improve selection of patients for standard-intensity therapy, attenuated therapy, or alternative approaches. Such work would help refine individualized treatment strategies for older adults while preserving the curative intent of frontline immunochemotherapy.

## 5 Conclusion

This study shows that in elderly patients with diffuse large B-cell lymphoma receiving first-line R-CHOP-based treatment, prognosis is influenced not only by tumor burden but also by treatment delivery and physiological reserve. Patients older than 80 years experienced substantially higher rates of severe infection, cardiac toxicity, and treatment-related mortality, indicating a markedly narrower therapeutic window in the very elderly population.

Among the evaluated variables, reduced relative dose intensity and hypoalbuminemia emerged as independent predictors of worse overall survival beyond conventional disease-based risk factors. These findings suggest that the ability to maintain adequate treatment intensity is a major determinant of outcome in older patients, and that baseline nutritional reserve has direct clinical relevance for survival.

Accordingly, frontline treatment decisions in elderly diffuse large B-cell lymphoma should move beyond uniform age-based dose reduction and toward risk-adapted strategies that integrate functional assessment, comorbidity burden, nutritional status, and treatment tolerance. For patients with adequate reserve, standardized treatment delivery supported by proactive toxicity management may help preserve curative potential. For frail or high-risk patients, individualized treatment modification and supportive care planning are essential to balance efficacy and safety.

## Data Availability

The raw data supporting the conclusions of this article will be made available by the authors, without undue reservation.
